# Increasing Isolation Efficiency Using a Segmented Quadrupole Mass Filter Operated with Rectangular Waveforms

**DOI:** 10.1021/jasms.4c00051

**Published:** 2024-04-30

**Authors:** Robert L. Schrader, Gordon A. Anderson, David H. Russell

**Affiliations:** † Department of Chemistry, Texas A&M University, College Station, Texas 77843, United States; ‡ GAA Custom Electronics, Kennewick, Washington 99338, United States

**Keywords:** quadrupole mass spectrometry, digital mass filter, Brubaker prefilters, Orbitrap mass spectrometry

## Abstract

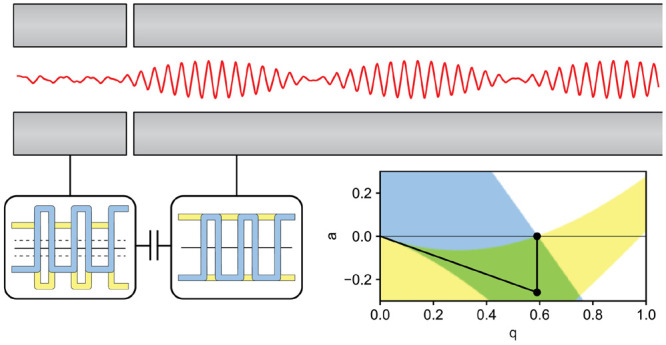

The performance of a segmented quadrupole mass filter operated with rectangular waveforms and capacitively coupled rectangular waveforms applied to the prefilters was examined on a home-built quadrupole-Orbitrap platform. For peak widths of 50 *m*/*z*, 100% isolation efficiency was achieved, which fell to approximately 20% for 5 *m*/*z* peak width for a rectangular waveform of 150 V_0–p_. Due to a small exit aperture following the mass filter, peak structure was observed in both experimental peak shapes and those simulated using SIMION. A larger radius quadrupole was examined and achieved similar performance. While the segmented quadrupole does remove the defocusing effects of the fringing fields, the ion beam is only slightly refocused due to the low RF voltage which limits achievable gains in isolation efficiency.

## Introduction

The quadrupole mass filter separates ions based on their stability within a radiofrequency quadrupolar field formed by four (ideally) hyperbolic electrodes.^1,2^ The stability of ions within the mass filter are defined in terms of Mathieu parameters *q* and *a*
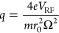
1
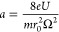
2where *e* is the elementary charge, *V*_RF_ is the zero to peak RF voltage, *m* is the mass of the ion, *r*_0_ is the rod radius, Ω is the angular frequency of the RF, and *U* is the 0 to rod DC voltage applied between the rod pairs. Most commonly, sinusoidal waveforms are applied to the rod pairs and selection is achieved by varying the RF and DC voltage amplitudes so that ions are near the apex of the stability diagram at *q* = 0.706, *a* = 0.23699 such that only a narrow band of *m*/*z* values will have stable trajectories.^3^ It is also possible to vary the stable *m*/*z* value by varying the drive frequency.^1,4,5^

For the digital quadrupole, the duty cycle can be changed which yields changes in the stability diagram^6−8^ from which a mass filter^9−16^ or ion trap^17−24^ can be constructed. For a square wave (50.0/50.0 duty cycle), the stability diagram is very similar to a sinusoidal quadrupole mass filter with a low mass cut off at *q* = 0.712 versus *q* = 0.908. Modulation of the duty cycle removes the need for additional DC power supplies by changing the stability diagram such that an adjustable window of *q* values is stable along the *a* = 0 axis.^6−8^

Fringing fields occur at the ends of the quadrupole rods where the ions experience the gradual strengthening or weakening of the quadrupolar field.^25−27^ Entering and exiting the quadrupole the ion traverses the mass scan line in Mathieu *a* and *q* space to the operating point of the mass filter. In both the sinusoidal and digital quadrupole mass filter, the ion is unstable in the *y* direction within the fringing fields.^28^ This leads to ion losses, especially for slow ions and ions of high *m*/*z* both spending many RF cycles in the fringing fields. The motion of ions within the fringing fields of a sinusoidal quadrupole has been well studied.^26,29−31^ To overcome the fringing fields, Brubaker added a short RF-only quadrupole adjacent to the mass filter where the RF field develops prior to the resolving DC field (delayed DC ramp).^25^ Many studies have been done calculating the trajectories of ions within a system with the delayed DC ramp.^26,32−35^ Other methods of separating the development of the RF and DC fields without separate RF electrodes have been developed.^36−38^ It is likely that many of the previously developed methods to overcome the fringing fields will be applicable to the digital mass filter.

While the resolving field in the digital mass filter is generated by the duty cycle and not a DC field, the principles of the delayed DC ramp can still be applied. A square waveform applied to the prefilter allows the ion to traverse the *a* = 0 axis within the stable region prior to entering the main rod section with a rectangular waveform which yields ion selection.^15,16,28^ This method requires four independent waveform outputs between the pre and postfilters and main rod sections. Alternatively, the rectangular waveforms can be directly applied to the main rod section and then capacitively coupled to the pre and postfilters,^12,39^ requiring only two independent waveform outputs. A sufficiently large capacitance will not distort the rectangular waveform and add a quadrupolar DC voltage offset such that *a* < 0.^14^

In this work, we investigate the performance of a segmented quadrupole mass filter operated with rectangular waveforms which were capacitively coupled to the pre and postfilters. Using a home-built digital quadrupole-Orbitrap mass spectrometer, quadrupole peak shapes were determined by scanning the digital quadrupole drive frequency at varying duty cycles yielding peak width vs isolation efficiency curves for the digital quadrupole mass filter. Comparisons between a 4 mm *r*_0_ and 5.25 mm *r*_0_ quadrupole yield insights into the challenges of operating a digital mass filter at low RF voltages.

## Experimental Section

### Chemicals

The Pierce LTQ Velos ESI positive Ion Calibration solution was purchased from Thermo Scientific (Rockford, IL). Borosilicate glass capillaries (10 cm length, 1.5 mm o.d., 0.86 mm i.d.) were purchased from Sutter Instrument (Navajo, CA). A Sutter Instrument P-1000 micropipette puller was used to pull the glass to 1–5 μm opening. The solution was pipetted into the pulled glass capillary and high voltage (1.1–1.5 kV) was applied through platinum wire (0.3 mm, Thermo Scientific).

### Instrumentation

**Figure 1:**
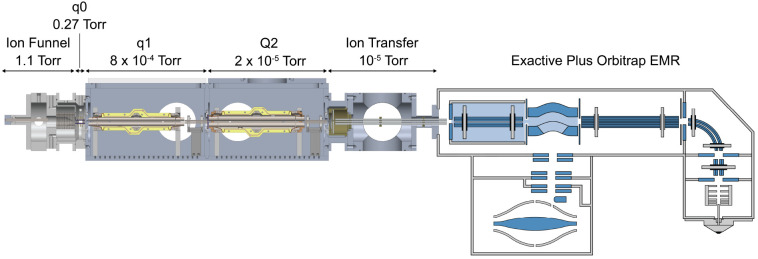
Solidworks rendering of the instrument interfaced with the rear of the HCD cell of an Exactive Plus Orbitrap EMR mass spectrometer. Modifications to the instrument include (i) shifting the heated capillary off the center axis, (ii) an extended length *q*0 to reduce the distance between the ion funnel exit/*q*0 entrance to increase ion activation, (iii) smaller *r*_0_ (2.75 mm) octopole ion guides, (iv) capacitively coupling the rectangular waveforms to the pre/postfilters to improve isolation efficiency, and (v) a Thermo Fisher QR5 segmented quadrupole mass filter operated with rectangular waveforms.

The instrument has been previously described in detail and is shown in Figure 1.^13,14^ Briefly, ions enter through a heated capillary (120 °C) into an RF ion funnel (472 kHz, 250 V_p–p_) region maintained at 1.1 Torr. The following vacuum region is maintained at 0.27 Torr by an additional mechanical pump and contains a 3.5 mm *r*_0_ planar segmented quadrupole ion guide (1.44 MHz, 500 V_p–p_). The following region is maintained at 8 × 10^–4^ Torr by a turbomolecular pump and contains a Thermo Fisher 4 mm *r*_0_ segmented quadrupole mass filter (1.44 MHz, 2500 V_p–p_) and a 2.75 mm *r*_0_ octopole ion guide (784 kHz, 450 V_p–p_). The following region contains the mass filter and is maintained at 2 × 10^–5^ Torr by an additional turbomolecular pump. Both a 4 mm *r*_0_ and 5.25 mm *r*_0_ Thermo Fisher quadrupole mass filter (Part Numbers 80100–60109 and 80133–60100, respectively) were tested. Both mass filters are segmented such that the initial and final 22 mm of the rods are separated structures, referred to as prefilters and postfilters, respectively. The rectangular waveform is applied directly to the main rods and capacitively coupled via 4000 pF capacitors to the pre and postfilters. The mass filter is followed by a 2.75 mm *r*_0_ octopole ion guide (786 kHz, 450 V_p–p_). Following an 800 μm skimmer aperture, the ions enter a 4.75 mm *r*_0_ octopole ion guide (902 kHz, 250 V_p–p_) to the exit of the HCD cell of a Thermo Fisher Exactive Plus Orbitrap EMR (Bremen, Germany). All RF and DC voltages were generated by Modular Intelligent Power Supplies (MIPS) systems and High Q RF heads (GAA Custom Electronics).

For operation of the digital quadrupole, high voltage waveforms were generated by two DEI PVX-4140 pulse generators (Directed Energy, Inc., Fort Collins, CO). Positive and negative voltage inputs were generated by two XP Power model PLS6004002.5 (Pangbourne, U.K.) DC power supplies. TTL signals of 50–500 kHz and arbitrary duty cycle were generated by an Astraea waveform generator (GAA Custom Electronics).

Acquisition of quadrupole mass spectra was automated using the Thermo Fisher Xcalibur and MIPS software packages. Xcalibur sequence files were generated with one ten second Orbitrap acquisition for each digital quadrupole frequency step. A script within the MIPS software swept the digital quadrupole frequency and enabled Orbitrap acquisition via a digital (5 V) output to the Orbitrap contact closure. Following acquisition, “.RAW” files were converted to “.mzxml” using MSconvert^40^ and analyzed using MATLAB (The Mathworks, Inc., Natick, MA) with the Bioinformatics toolbox.

### Digital Quadrupole Simulations

Quadrupole peak shapes were simulated using ion trajectory simulations conducted in SIMION 8.1 (Scientific Instrument Services, Ringoes, NJ). The model consisted of the entrance lens and a Thermo Fisher 4 mm *r*_0_ segmented quadrupole mass filter or a Thermo Fisher 5.25 mm *r*_0_ segmented quadrupole mass filter. Geometries were generated with a grid size of 0.1 mm per grid unit with fractional surfaces. Ion initial conditions were *m*/*z* 1421.95, 1.5 mm beam diameter (circular distribution), 4.25 ± 0.5 eV kinetic energy (Gaussian distribution), and a 5° half angle beam divergence (Gaussian distribution), and 1000 ion trajectories were calculated for each *q* value. Both the kinetic energy and beam divergence were Gaussian distributions. The experimental ion kinetic energy was determined from a stopping curve. The quadrupole drive frequency was calculated from the input *q* value, ion mass, ion charge, 4 mm or 5.25 mm rod radius, and an RF voltage of 150 V_0–p_ and converted to waveform period in microseconds. Using the defined duty cycle, the periods for +V_RF_ and −V_RF_ were calculated in microseconds. By comparing the ion flight time in microseconds to the number of RF cycles elapsed, appropriate RF potentials were applied. An adjustable time step ensures that a time step occurred exactly at the waveform switch time.

## Results and Discussion

### Reducing Ion Kinetic Energy

The resolving power of the quadrupole mass filter decreases with increasing kinetic energy as the ions do not spend enough RF cycles in the mass filter.^3^ With the previous instrument design, the ions would enter the quadrupole with kinetic energies of at least 15–25 eV/charge. Adjusting the DC voltages to transmit lower kinetic energies resulted in little to no ion intensity. For a digital quadrupole operated at 150 V_0–p_, *r*_0_ of 4 mm, and quadrupole length of 203 mm, this corresponds to only 35–45 RF cycles in the mass filter. Note that for a frequency-modulated digital quadrupole mass filter the number of RF cycles is *m*/*z*-independent, unlike the sinusoidal quadrupole where higher *m*/*z* ions spend more cycles in the quadrupole. A derivation of the number of RF cycles spent in the mass filter is provided in the Supporting Information. As the kinetic energy was unaffected by the DC voltage settings, it was hypothesized that the excess kinetic energy was a result of the flowing gas from the heated capillary as the capillary was coaxial with the central axis and the ion funnel is only 48 mm in length.^41^

To disrupt the directed gas flow, the heated capillary was moved off the central axis by 0.375 times the radius of the first ion funnel electrode (Figure S1). This corresponds to approximately 4.76 mm compared with a final funnel electrode diameter of 2 mm. This removed the line-of-sight of the heated capillary to the later regions of the instrument. Following this change, ion kinetic energies were greatly reduced (Figure S2). The minimum kinetic energy where high signal intensity could be achieved was approximately 4.25 eV for the Ultramark tune mix ions. This corresponds to 83 RF cycles spent in the quadrupole, a factor of approximately two more than the high kinetic energy ions. The *q*1 region is maintained at approximately 8 × 10^–4^ Torr and contains a segmented quadrupole mass filter which has no DC potential along the axis to drive the ions axially as they are collisionally cooled. Therefore, it is possible that replacing the mass filter with a quadrupole with a supplementary axial field gradient would allow for transmission of ions with even lower kinetic energies and further increase the number of RF cycles the ions spend in the quadrupole. The ion kinetic energy is constant with any activating potential into *q*0, which suggests that the ions are completely collisionally cooled within *q*0. The remaining excess kinetic energy is hypothesized to be due to the gas flow from *q*0 (0.27 Torr) into *q*1 (8 × 10^–4^ Torr). As the number of RF cycles has an inverse square root relationship with kinetic energy, further reduction in the kinetic energy will yield a large increase in the number of RF cycles.

### Prefiltering with Capacitively Coupled Rectangular Waveforms

**Figure 2:**
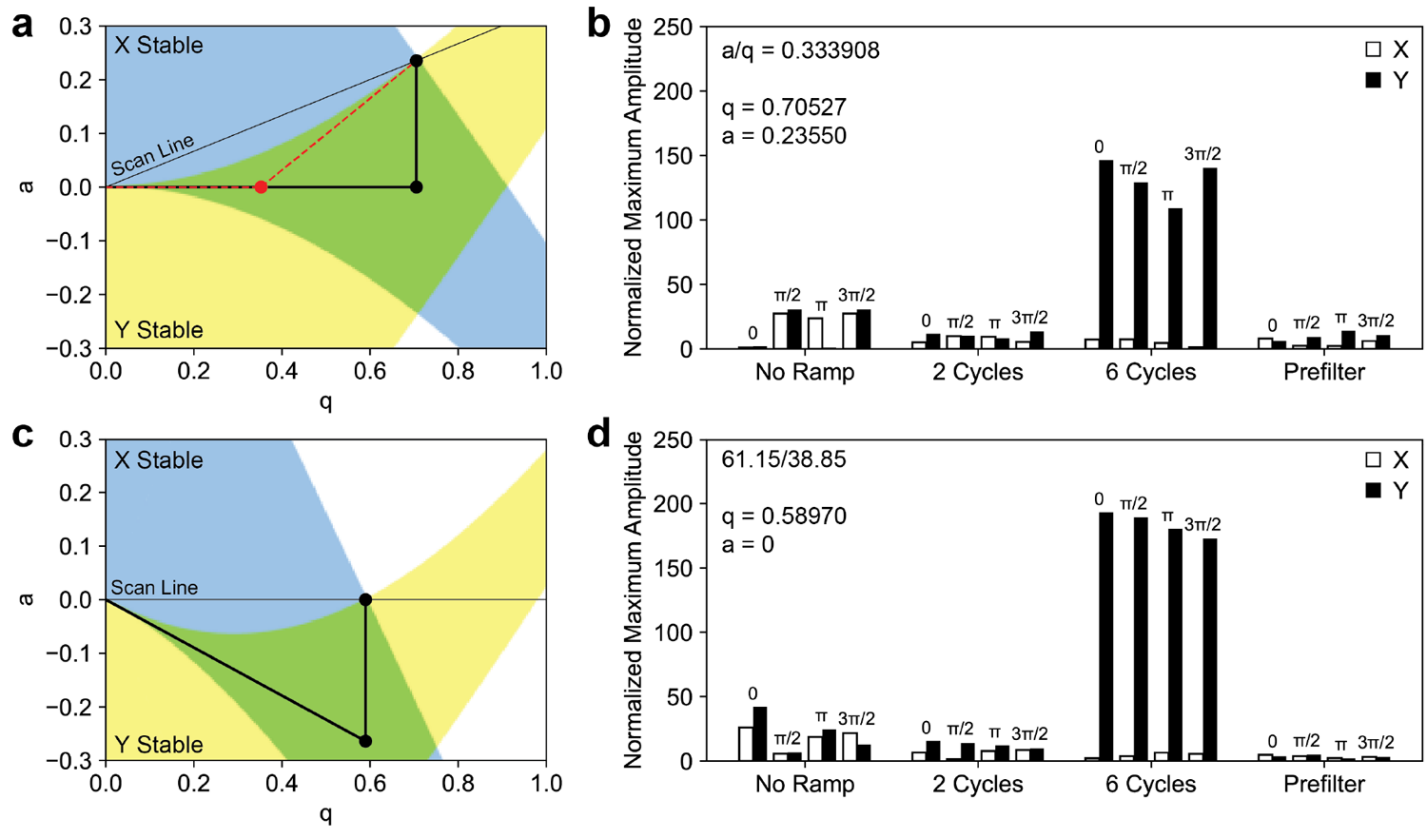
(a) Stability diagram for a sinusoidal mass filter showing the path of the ion to the operating point. Without a prefilter, the ion follows the scan line through a region of only *x* stability to the operating point. With an RF only prefilter, the *q* value increases prior to the *a* value. For comparison with digital operation, the *q* value of the prefilter and main rod are the same, though often only a portion of the RF is applied to the prefilter (shown in red). (b) Calculated maximum amplitudes for an ion spending various numbers of RF cycles in the fringing fields for a sinusoidal mass filter. (c) Stability diagram for a digital mass filter showing the path of the ion to the operating point. Analogous to the sinusoidal mass filter, the ion follows a path of only *x* stability without a prefilter. By capacitively coupling the rectangular waveform, the *a* value moves the ion to the *x* and *y* stable region through the prefilter. (d) Calculated maximum amplitudes for an ion spending various numbers of RF cycles in the fringing field for a digital mass filter.

Mirroring the calculations of Brubaker,^25^ matrix methods were used to calculate the maximum displacement of an ion beginning at *x* = *y* = 1 with velocity parallel to the ion axis for both sinusoidal and digital operation.^26,28,42^ The working point was chosen such that that RP_BL_ = 143 and β_*x*_ + β_*y*_ = 1. This corresponds to *q* = 0.70527, *a* = 0.23550 for a sinusoidal mass filter and *q* = 0.58970 with a duty cycle of 61.15/38.85 for a digital mass filter. The ion position and velocity were calculated over 50 RF cycles. With no RF ramp, the *q* value was at the operating point for the entire calculation. For sinusoidal operation, the fringing fields were modeled as a linearly increasing *q* and *a* value from 0 over a defined number of RF cycles. For digital operation, the *q* value was increased with constant *a* value and duty cycle (Figure 2).

As observed in calculations by both Brubaker^25^ and Dawson,^26^ the maximum amplitude of the ion trajectory is decreased for a short number of cycles in the fringing fields for both sinusoidal and digital operation when compared with no fringing fields. As the number of cycles spent in the fringing field increases, the ion spends more time in the region of *y* instability and the *y* displacement increases greatly for both sinusoidal and digital operation resulting in ion losses for both modes of operation. Interestingly, the maximum amplitude for digital operation is approximately 1.4 times larger than sinusoidal for an ion spending six RF cycles in the fringing fields. For the unstable *y* trajectories, the parameter μ, or the increment of exponential growth of the ion oscillation over one RF cycle, can be considered^7^ (Figure S3). Much like the stability diagram for square wave operation is compressed over the *q* axis when compared with sinusoidal operation, the same is true for the parameter μ.

To model ions entering the quadrupole through a prefilter, the ions spent two RF cycles entering the prefilter, five RF cycles within the prefilter, two RF cycles exiting the prefilter, and two RF cycles entering the main rod. Commonly, the RF applied to the prefilter is coupled from the RF to the main rod through a small value capacitor which results in a percentage of the full RF voltage being applied to the prefilter. This path is shown as a dotted red line in Figure 2. For comparison with digital operation where the *q* value in the prefilter is the same as the main rod, the full *q* value was used in the prefilter. For digital operation, the rectangular waveform is applied directly to the main rod and then capacitively coupled through a 4000 pF capacitor. With this capacitance, the waveform maintains its square. As a capacitor can only pass a time-averaged voltage of 0, when the rectangular waveforms area capacitively coupled they are offset by a DC voltage to yield a time-averaged voltage of zero.^14^ This results in a quadrupolar DC offset in the prefilter such that *q* = 0.5897, *a* = −0.2640. Analogous to the sinusoidal quadrupole, the path of the ion to the operating point is only through the *x* and *y* stability region. The advantages of prefiltering for both sinusoidal and digital quadrupole operation can be seen from the reduction in maximum ion amplitudes.

### Isolation Efficiency of a Segmented Digital Quadrupole Mass Filter

To determine the isolation efficiency, quadrupole mass spectra were collected by plotting Orbitrap extracted ion intensities versus *m*/*z* which is calculated from the quadrupole drive frequency. To calculate isolation efficiency, the ion intensities for the selected ion were compared with the ion intensity from an Orbitrap full scan with the digital quadrupole set to a 50.0/50.0 duty cycle and 500 kHz drive frequency. This corresponds to an *m*/*z* 514 (*q* = 0.712) low mass cut off and is analogous to the RF-only mode of the sinusoidal quadrupole. Representaitve Orbitrap mass spectra are shown in Figure S4.

**Figure 3:**
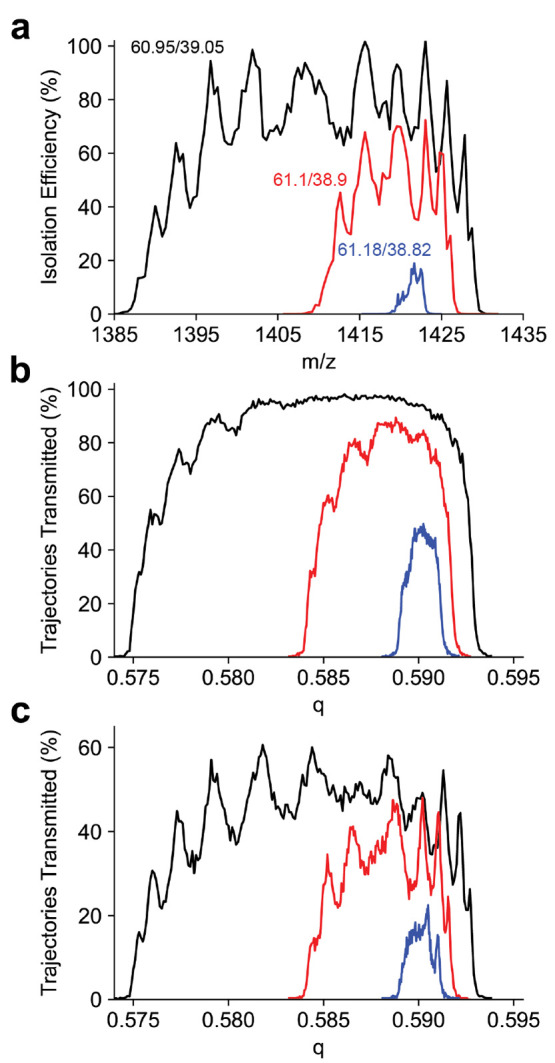
(a) Experimental quadrupole peak shapes at duty cycles 60.95/39.05, 61.1/38.9, and 61.18/38.82 (corresponding to theoretical resolving power of 33, 78, and 287, respectively) for the *m*/*z* 1422 Ultramark ion with the 4 mm *r*_0_ quadrupole. (b) Simulated quadrupole peak shapes for the same duty cycles. (c) Simulated quadrupole peak shapes for the same duty cycles for only ions having a diameter less than the exit skimmer diameter of 800 μm. The experimentally observed peak splitting is reporduced in the simulation and is a result of the varying diameter of the ion beam exiting the quadrupole mass filter.

Peak shapes for the *m*/*z* 1422 ion are shown in Figure 3a. For each resolving power the peak shape is split, as previously observed for sinusoidal quadrupoles.^43−45^ To investigate this peak splitting, the peak shape was simulated using SIMION. The initial kinetic energy distribution was chosen based on the experimentally determined kinetic energy (Figure S2). The peak shapes generated from trajectory simulations also have a structure to the low *q* side of the peak, but do not reproduce the splitting observed in the experimental peak shapes (Figure 3b). As the SIMION model only included the entrance lens and the quadrupole mass filter, this simulation does not account for the octopole ion guide or 800 μm skimmer aperture that follow the mass filter. As the pressure in the quadrupole chamber is approximately 2 × 10^–5^ Torr, we assume the beam dimensions do not change through the octopole ion guide due to collisional cooling. Therefore, to account for this aperture, all trajectories with a final diameter in either the *x* or *y* dimension larger than 800 μm were removed. This results in an approximately 60% reduction in transmission efficiency, and the peak splitting observed in the simulated peak shape reproduces what is observed experimentally (Figure 3c). Previous experiments with C-reactive protein (*m*/*z* 4800) did not show a similar peak profile.^14^ Several modifications to the ion optics may contribute to the peak splitting now observed. Previously, the octopole ion guide prior to the quadrupole mass filter had an inscribed radius of 4.5 mm and maintained the 2 mm quadrupole entrance aperture diameter. This caused many ions to be lost to the entrance aperture due to the large beam dimension. The octopole ion guide following the mass filter had an inscribed radius of 3.5 mm, leaving ions spread by the mass filter to be lost to the exit aperture. Both octopole ion guides were replaced with ones with an inscribed diameter of 2.75 mm. This results in better beam quality entering and exiting the mass filter which greatly reduces ion losses. Ion losses lead to a cropping of the top of the peak where any ions reaching a large displacement from the central axis were lost masking any observable peak splitting.

**Figure 4:**
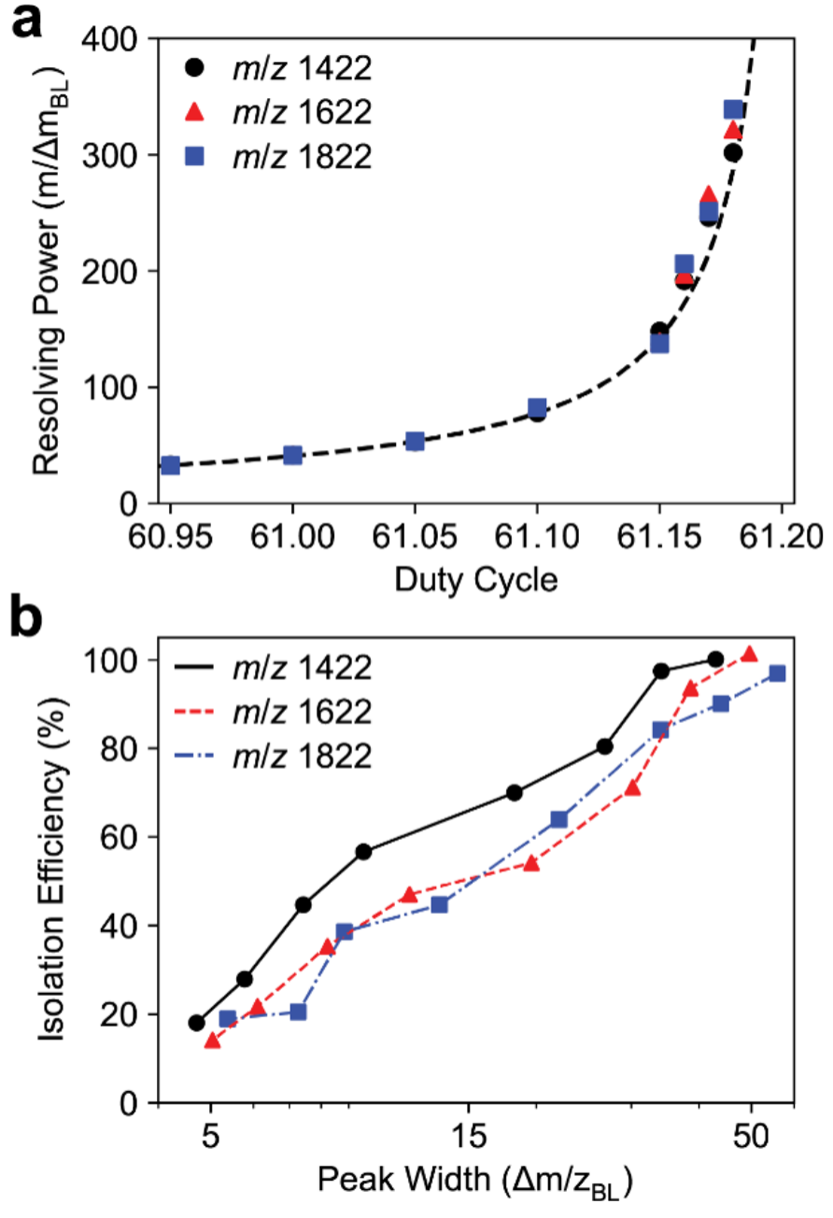
(a) Baseline resolving power (*m*/Δ*m*) for the *m*/*z* 1422, 1622, and 1822 Ultramark ion versus duty cycle with the 4 mm *r*_0_ quadrupole. The dashed line corresponds to the theoretical resolving power (*q*/Δ*q*) calculated from the stability diagram. (b) Quadrupole isolation efficiency for the *m*/*z* 1422, 1622, and 1822 Ultramark ions for varying peak widths with the 4 mm *r*_0_ quadrupole. The isolation efficiency is ion intensity relative to the intensity of the ion in an Orbitrap full scan with the quadrupole mass filter set to 500 kHz and a 50.0/50.0 duty cycle.

The isolation efficiency of the quadrupole mass filter is inversely related to the peak width. Quadrupole peak shapes were measured for three Ultramark ions (*m*/*z* 1422, 1622, and 1822). The theoretical resolving power is calculated from *q*/Δ*q* of the stable *q* values on the *a* = 0 axis for a given duty cycle. Experimentally measured baseline resolving powers show good agreement with theoretical values (Figure 4a). Isolation for each Ultramark ion for a 50 *m*/*z* peak width was approximately 100% and decreased to approximately 20% for a 5 *m*/*z* peak width (Figure 4b). Interestingly, the *m*/*z* 1622 and 1822 ions have a slightly reduced isolation efficiency at each peak width compared with the *m*/*z* 1422 ion.

### Increasing the Quadrupole Field Radius

The quadrupole acceptance area is inversely proportional to the square of the drive frequency,^3^ though the relationship to signal intensity is more complex due to the matching of the ion beam to the quadrupole acceptance area.^46^ The acceptance area is also directly proportional to *r*_0_^4^, therefore it would be advantageous to use a quadrupole with a larger field radius. The field radius of the quadrupole mass filter of the Thermo Fisher Orbitrap Eclipse Tribrid mass spectrometer was increased from 4 mm to 5.25 mm.^47,48^ By increasing the field radius, acceptance is increased which results in higher isolation efficiency, particularly at high *m*/*z*.^47^ No additional modifications were required to replace the 4 mm *r*_0_ quadrupole with the 5.25 mm *r*_0_ quadrupole. As the field radius is increased, the ion *q* values are reduced for identical voltage and drive frequency conditions. To maintain consistent *q* values between the two quadrupoles, the drive frequency for a full scan was reduced from 500 kHz to 380.952 kHz. Alternatively, the RF voltage could be increased from 150 V_0–p_ to 258.4 V_0–p_. This was not chosen due to the increase in power demand for the waveform generators. This means that for the 5.25 mm *r*_0_ quadrupole, all the isolation frequencies are reduced by a factor of 1.3125. Therefore, the ions spend less RF cycles in the 5.25 mm *r*_0_ quadrupole than the 4 mm *r*_0_ quadrupole. While the ions spend 88 RF cycles in the 4 mm *r*_0_ quadrupole with a kinetic energy of 4.25 eV, only 67 RF cycles are spent in 5.25 mm *r*_0_ quadrupole due to the reduced drive frequency. Example ion trajectories showing the reduced number of RF cycles are shown in Figure S5).

**Figure 5:**
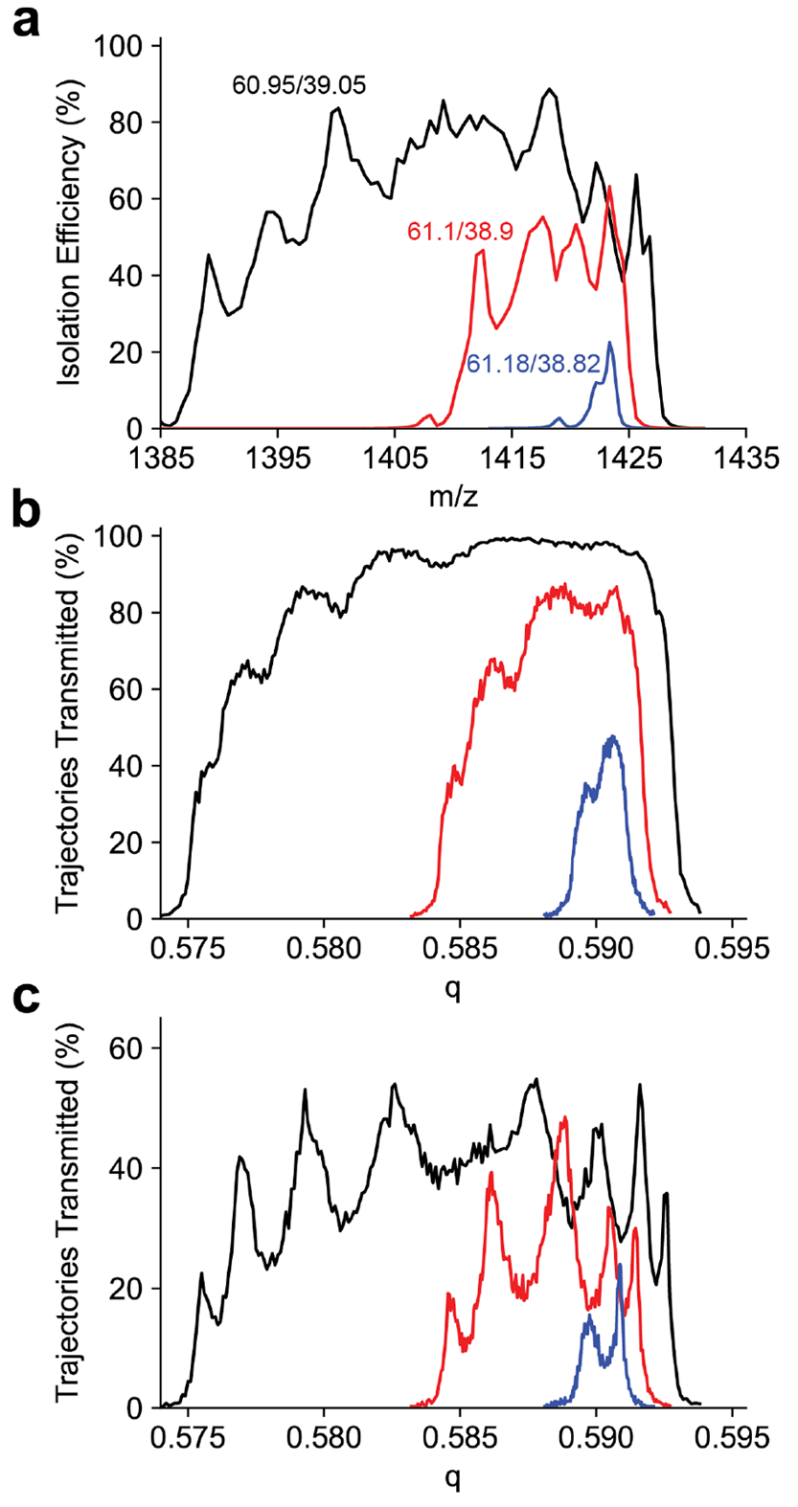
(a) Experimental quadrupole peak shapes at duty cycles 60.95/39.05, 61.1/38.9, and 61.18/38.82 for the *m*/*z* 1422 Ultramark ion with the 5.25 mm *r*_0_ quadrupole. (b) Simulated quadrupole peak shapes for the same duty cycles. (c) Simulated quadrupole peak shapes for the same duty cycles for only ions having a diameter less than the exit skimmer diameter of 800 μm. As the drive frequency is reduced for these isolations, the number of nodes in the peak is lower when compared to the 4 mm *r*_0_ quadrupole.

Like the 4 mm *r*_0_ quadrupole, experimental and simulated peak shapes for *m*/*z* 1422 ion are shown for the 5.25 mm *r*_0_ quadrupole (Figure 5). The effect of the reduced drive frequency is reflected in the reduced number of nodes on the top of the peak. Interestingly, a small extraneous peak can be seen on the low *m*/*z* side of the peak. This was also observed for the *m*/*z* 1622 and 1822 ions at each resolving power studied but is not seen with the 4 mm *r*_0_ quadrupole. This feature is not yet understood and requires further study.

**Figure 6:**
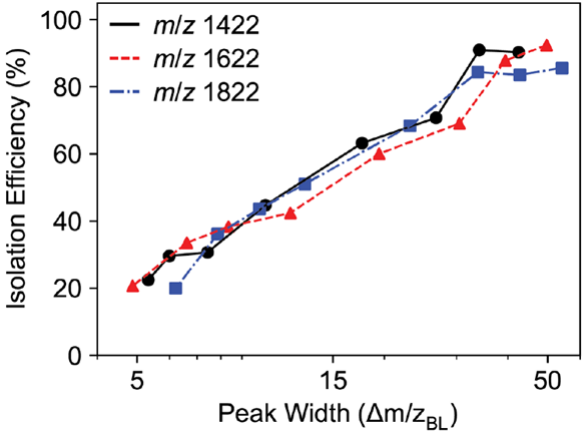
Quadrupole isolation efficiency for the *m*/*z* 1422, 1622, and 1822 Ultramark ions for varying peak widths with the 5.25 mm *r*_0_ quadrupole. The isolation efficiency is ion intensity relative to the intensity of the ion in an Orbitrap full scan with the quadrupole mass filter set to 380.952 kHz and a 50.0/50.0 duty cycle. The frequency was chosen to keep consistent *q* values between the 4 mm and 5.25 mm *r*_0_ quadrupole.

The increased acceptance of the larger quadrupole can be seen from a plot of signal intensity versus *q*1 RF amplitude. For the 4 mm *r*_0_ quadrupole, the signal intensity is nearly linear with *q*1 RF amplitude while for the 5.25 mm *r*_0_ quadrupole the signal plateaus at lower RF amplitudes (Figure S6). While the increased acceptance was observed, the isolation efficiency did not improve, and is even reduced for a 50 *m*/*z* peak width (Figure 6). The difference in isolation efficiency between the three ions was less for the 5.25 mm *r*_0_ quadrupole compared to the 4 mm *r*_0_ quadrupole. Because the acceptance area is larger for the 5.25 mm *r*_0_ quadrupole, it will be less probable to observe changes in ion intensity based on the drive frequency.

**Figure 7:**
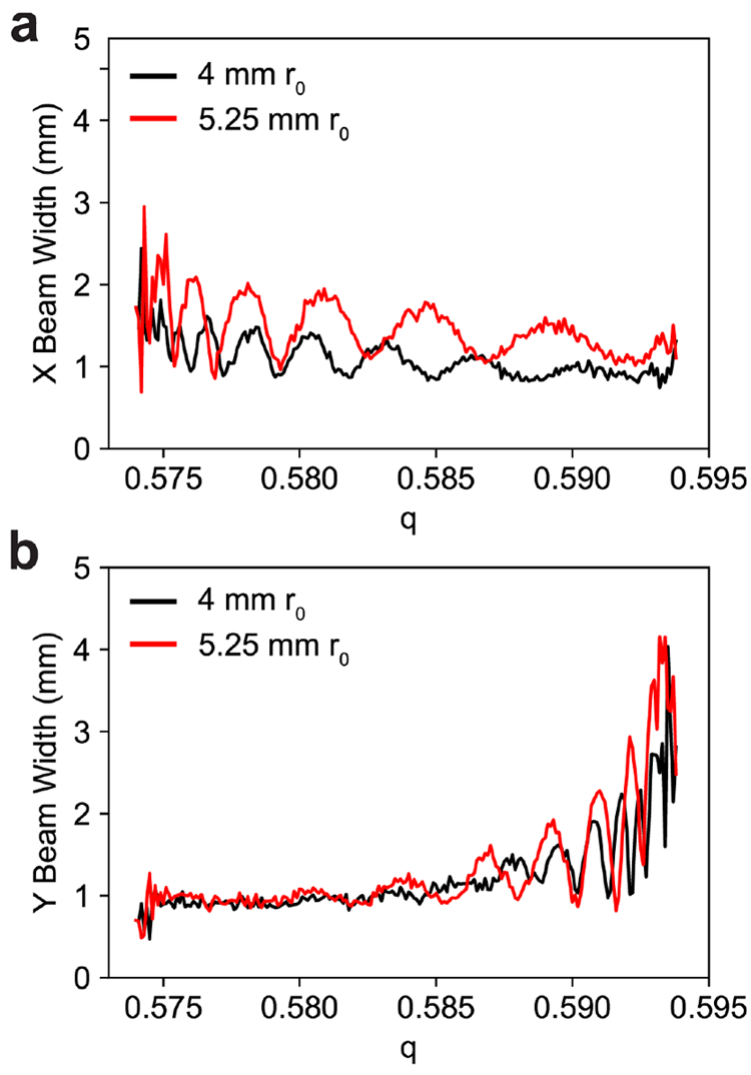
Simulated ion beam widths (at fwhm) for a 60.95/39.05 duty cycle, *q* = 0.5839, and *V*_RF_ = 150 V for a 4 mm *r*_0_ quadrupole and 5.25 mm *r*_0_ quadrupole in the (a) *X* dimension and (b) *Y* dimension. The low RF voltage of the postfilter does not strongly focus the ion beam resulting in large beam dimensions that are easily lost to the exit aperture.

While the isolation efficiency for 50 *m*/*z* peak width is approximately 100% for the 4 mm *r*_0_ quadrupole, it is only about 90% for the 5.25 mm *r*_0_ quadrupole. Comparing the intensities of the simulated peak shapes, SIMION correctly predicts this effect. Comparing the width of the ion beam between the 5.25 mm and 4 mm *r*_0_ quadrupole, the ion beam exiting the larger quadrupole is larger leading to more ion losses on the skimmer aperture(Figure 7). While the ions have stable trajectories in the postfilter, the RF voltage is only 150 V_0–p_, unlike a sinusoidal quadrupole where this voltage would be far larger. This limits the ability of the postfilter to refocus the ion beam after the ions are spread by the mass filter. For smaller peak widths, the larger quadrupole does begin to outperform the smaller quadrupole. For high resolving powers, ions are more prone to be lost in the beginning of the mass filter which benefits from the larger field radius.

## Conclusions

Capacitively coupling rectangular waveforms to the pre and postfilter of a segmented quadrupole mass filter improves isolation efficiency for the digital quadrupole mass filter. Using the Ultramark *m*/*z* 1422, 1622, and 1822 ions, the mass filter was able to achieve approximately 100% isolation efficiency for a 50 *m*/*z* peak width and approximately 20% isolation efficiency for a 5 *m*/*z* peak width. Significant peak splitting was observed due to the small 800 μm aperture that follows the mass filter. Using a larger 5.25 mm *r*_0_ quadrupole compared to a 4 mm *r*_0_ quadrupole did not yield large improvements in performance. Low operating voltages, while advantageous to power consumption of the waveform generators, limits the refocusing of the ion beam in the postfilter and therefore achievable performance. For low operating voltages, a tightly focused ion beam into and out of the mass filter reduces the chance ions will be lost to the quadrupole rods.

## Supporting Information Available

This material is available free of charge at . The Supporting Information is available free of charge at https://pubs.acs.org/doi/10.1021/jasms.4c00051.

Derivation of the number of RF cycles in a digital mass filter; modifications to ion funnel source design; stopping curves to determine ion kinetic energy; comparison of the fringing fields in a sinusoidal versus digital mass filter; representative full scan and isolated Orbitrap mass spectra; example SIMION ion trajectories for the 4 mm and 5.25 mm *r*_0_ quadrupole; signal intensity as a function of *q*_1_ RF voltage for the 4 mm and 5.25 mm *r*_0_ quadrupole (PDF)
