# Supplementary material for: Increasing Isolation Efficiency Using a Segmented Quadrupole Mass Filter Operated with Rectangular Waveforms
Source: J Am Soc Mass Spectrom. Author manuscript; Available in PMC 2024 Jun 6. (PMC11153046; doi:10.1021/jasms.4c00051)
Supplement: published SI [file NIHMS1992579-supplement-published_SI.pdf]

Supporting Information

Increasing Isolation Efficiency Using a Segmented Quadrupole Mass Filter Operated with Rectangular Waveforms

Robert L. Schrader[1], Gordon A. Anderson[2], and David H. Russell[1,*]

[1]Department of Chemistry, Texas A&M University, College Station, TX 77843
[2]GAA Custom Electronics, Kennewick, Washington 99338, United States

*Corresponding author; email: russell@chem.tamu.edu

Table of Contents

	Page
Derivation of the Number of RF Cycles in a Digital Mass Filter	S2
Figure S1	S3
Figure S2	S4
Figure S3	S5
Figure S4	S6
Figure S5	S7
Figure S6	S8

Derivation of the Number of RF Cycles in a Digital Mass Filter

The ion's transit time through the quadrupole can be calculated from the ion's axial kinetic energy, where z is the number of charges on the ion, e is the charge of an electron, V is the axial accelerating potential, m is the mass of the ion, and v is the ion velocity.

$$zeV = \frac{1}{2}mv^2$$

By substituting the length of the quadrupole (L) and transit time (t) for velocity, the transit time is calculated:

$$zeV = \frac{1}{2}m\left(\frac{L}{t}\right)^2$$

$$t = L\sqrt{\frac{m/z}{2eV}}$$

Dividing the transit time by the period of the RF waveform gives the transit time in RF cycles (n_{RF}). To determine the period, the RF drive frequency is calculated from Mathieu q

$$q = \frac{4eV_{RF}}{m/z\, r_0^2 \Omega^2}$$

where V_{RF} is the 0 to peak amplitude of the RF, r_0 is the quadrupole field radius, and Ω is the angular frequency ($\Omega = 2\pi F$). After solving for F

$$n_{RF} = \frac{L\sqrt{\frac{m/z}{2eV}}}{1\Bigg/\frac{\sqrt{\frac{4eV_{RF}}{m/z\, r_0^2 q}}}{2\pi}}$$

$$n_{RF} = \frac{L}{2\pi}\sqrt{\frac{m/z}{2eV}\frac{4eV_{RF}}{m/z\, r_0^2 q}}$$

$$n_{RF} = \frac{L}{2\pi}\sqrt{\frac{2V_{RF}}{V r_0^2 q}}$$

For a digital mass filter operated in the first stability $q \approx 0.59$. Unlike a sinusoidal quadrupole where drive frequency is constant, the number of RF cycles is independent of m/z in the digital mass filter.

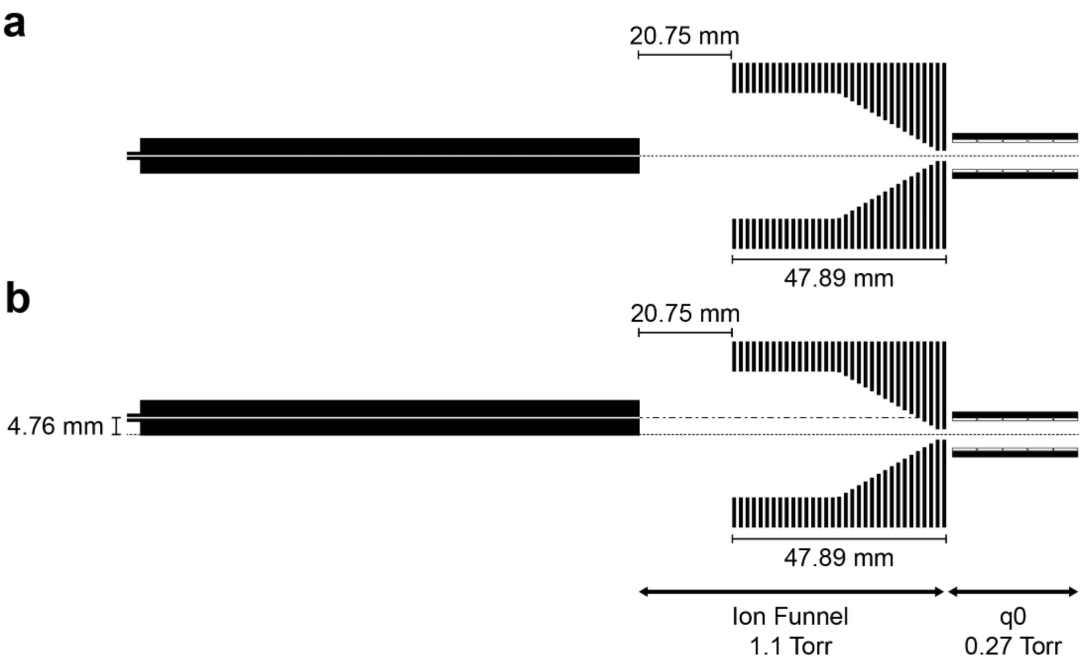

Figure S1. (a) Original ion funnel design with the heated capillary coaxial with the ion funnel axis. (b) Modified ion funnel design with the heated capillary offset by 0.375x the radius of the first funnel electrode to eliminate the line-of-sight into further vacuum regions.

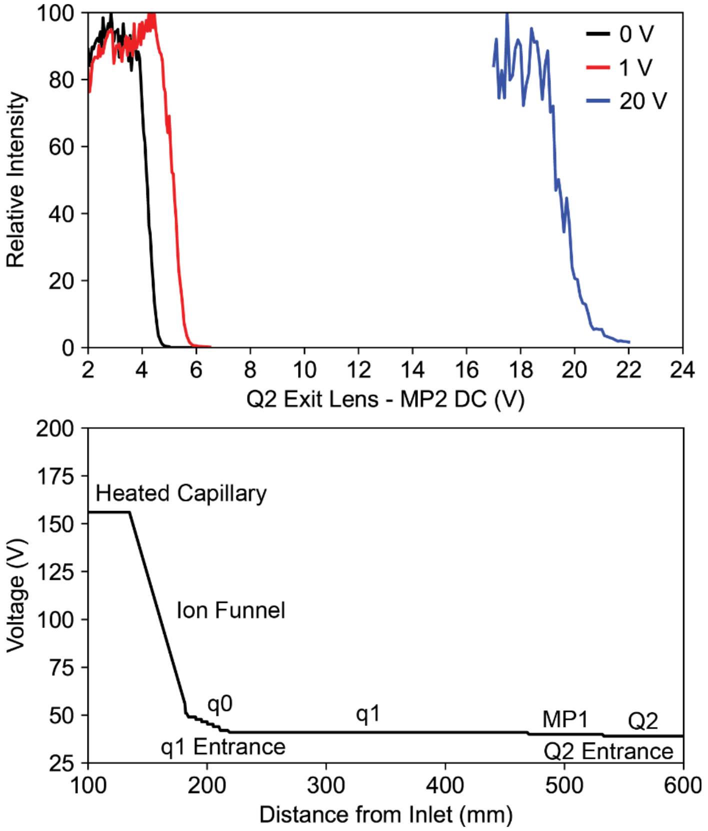

Figure S2. Stopping curves for various voltage differences between MP1 DC and the Q2 entrance lens DC. The ion kinetic energy varied from 4.25 eV for 0 V, 5.25 eV for 1 V, to 19.7 eV for 20 V. All other inter-optic voltage differences were kept constant.

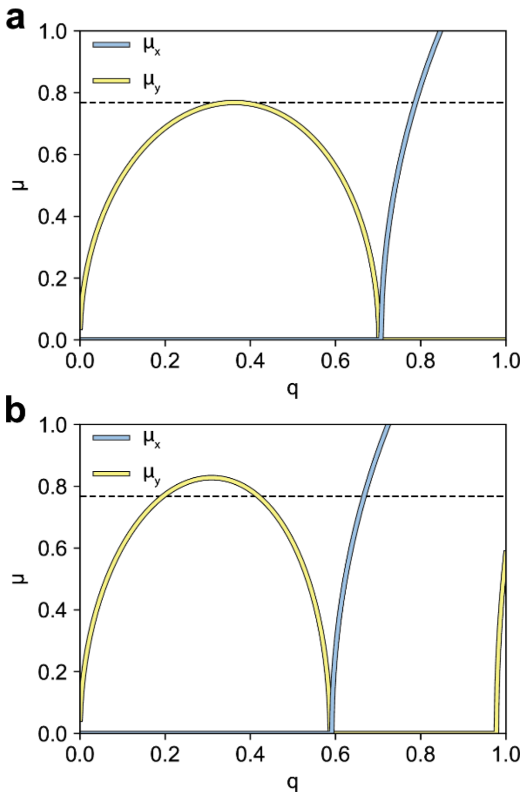

Figure S3. The parameter μ, the increment of exponential growth of the ion oscillation over one RF cycle, in the *x* and *y* direction for (a) sinusoidal and (b) digital operation. The maximum for the sinusoidal quadrupole is plotted as a horizontal line.

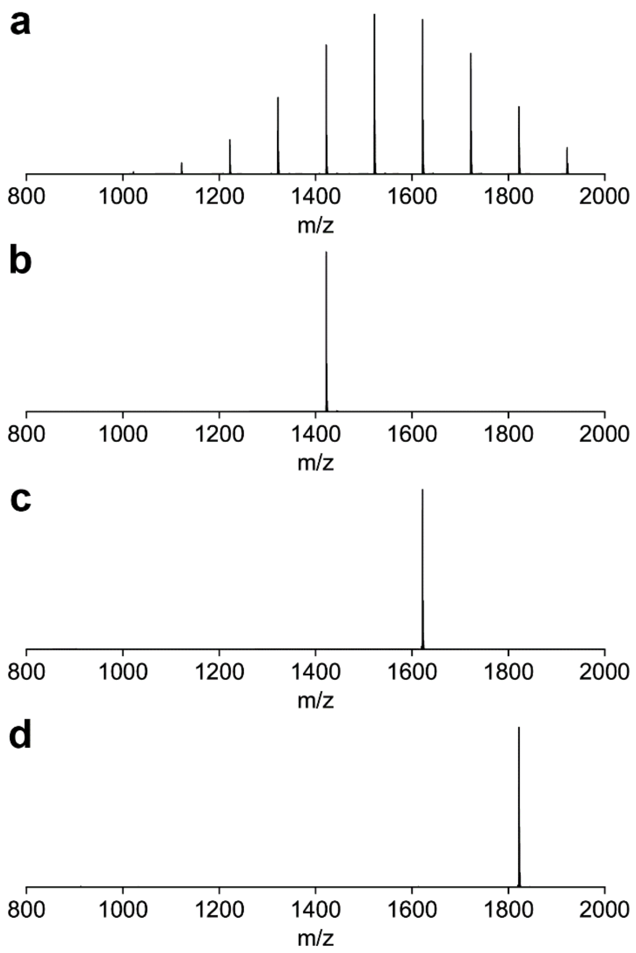

Figure S4. (a) Representative full scan Orbitrap mass spectrum with acquired with a 50.0/50.0 duty cycle. Representative Orbitrap spectra for isolations with a 60.95/39.05 duty cycle for (b) *m*/*z* 1422, (c) *m*/*z* 1622, and (d) *m*/*z* 1822.

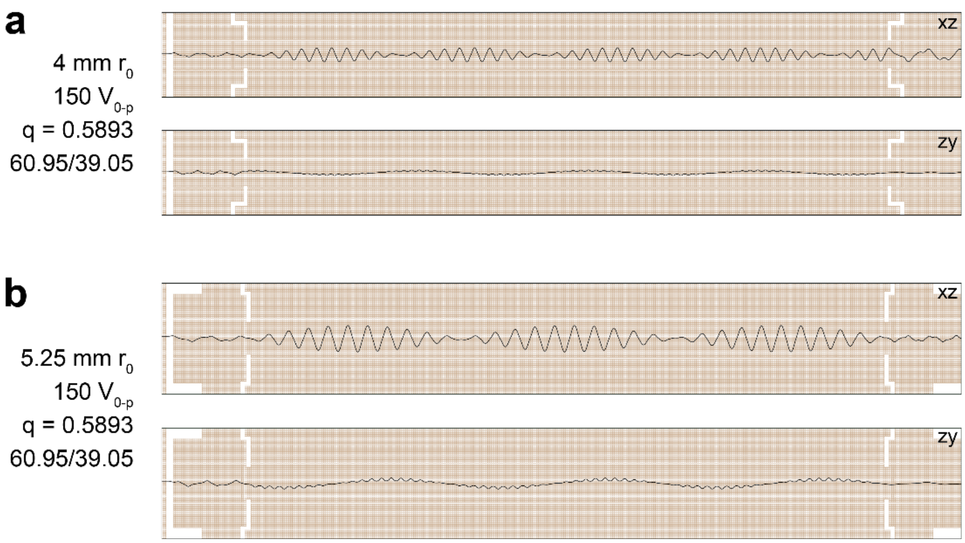

Figure S5. Example SIMION calculated ion trajectories in both the *xz* and *zy* plane for the (a) 4 mm r_0 mass filter and the (b) 5.25 mm r_0 mass filter. The decrease in the number of RF cycles that the ions experience in the 5.25 mm r_0 mass filter can be seen as the drive frequency is reduced.

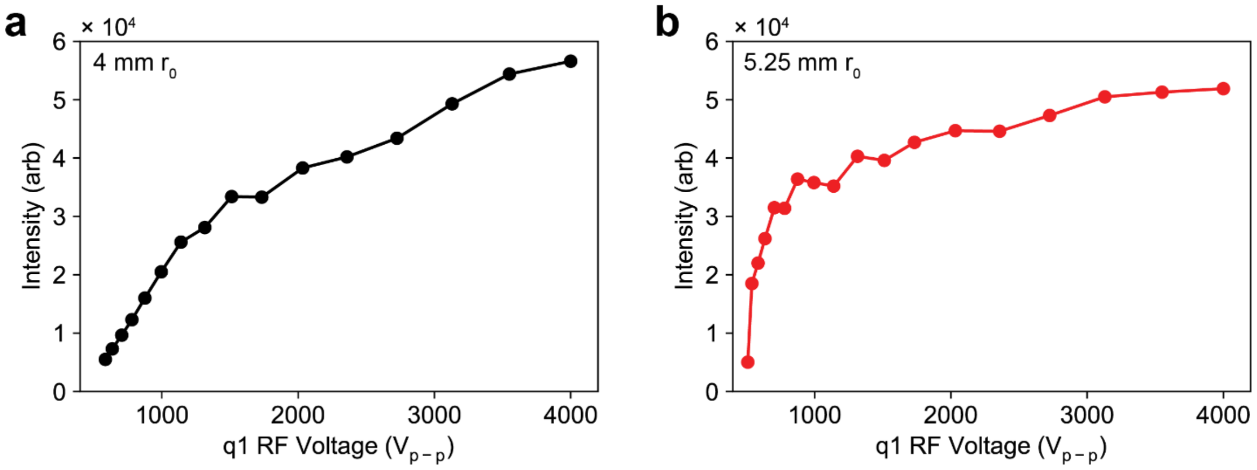

Figure S6. Orbitrap intensity for the m/z 1421.97 tune mix ion as a function of q1 RF voltage with a (a) 4 mm r_0 and a (b) 5.25 mm r_0. The RF voltage of the octopole ion guide between q1 and Q2 was maintained at a constant RF voltage of 450 $V_{p\text{-}p}$. The intensities between the two plots are not comparable as they are from different nanoelectrospray tips with different full scan intensities.
